# Iodine and plant-based diets: a narrative review and calculation of iodine content

**DOI:** 10.1017/S0007114523001873

**Published:** 2024-01-28

**Authors:** Katie Nicol, Anne P. Nugent, Jayne V. Woodside, Kathryn H. Hart, Sarah C. Bath

**Affiliations:** 1 Department of Nutritional Sciences, Faculty of Health and Medical Sciences, University of Surrey, Guildford, GU2 7XH, UK; 2 Institute for Global Food Security, School of Biological Sciences, Queens University Belfast, Northern Ireland, UK; 3 Centre for Public Health, Queens University Belfast, Belfast, UK

**Keywords:** Iodine, Plant-based, Vegan, Pregnancy, Milk, Fish

## Abstract

An increasing number of food-based recommendations promote a plant-based diet to address health concerns and environmental sustainability in global food systems. As the main sources of iodine in many countries are fish, eggs and dairy products, it is unclear whether plant-based diets, such as the EAT-Lancet reference diet, would provide sufficient iodine. This is important as iodine, through the thyroid hormones, is required for growth and brain development; adequate iodine intake is especially important before, and during, pregnancy. In this narrative review, we evaluated the current literature and estimated iodine provision from the EAT-Lancet reference diet. There is evidence that those following a strict plant-based diet, such as vegans, cannot reach the recommended iodine intake from food alone and are reliant on iodine supplements. Using the EAT-Lancet reference diet intake recommendations in combination with iodine values from UK food tables, we calculated that the diet would provide 128 μg/d (85 % of the adult recommendation of 150 μg/d and 51–64 % of the pregnancy recommendation of 200–250 μg/d). However, if milk is replaced with unfortified plant-based alternatives, total iodine provision would be just 54 μg/d (34 % and 22–27 % of the recommendations for adults and pregnancy, respectively). Plant-based dietary recommendations might place consumers at risk of iodine deficiency in countries without a fortification programme and where animal products provide the majority of iodine intake, such as the UK and Norway. It is essential that those following a predominantly plant-based diet are given appropriate dietary advice to ensure adequate iodine intake.

## Graphical abstract

**Figure:**
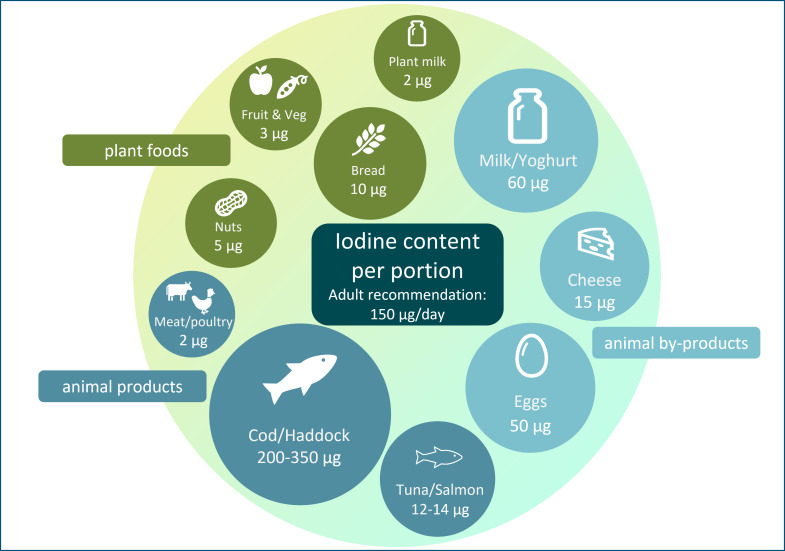

There is growing evidence that our current food system faces increasing environmental and health challenges. Global food production is responsible for about one-third of all greenhouse gas emissions while threatening climate stability and ecosystem resilience^(1)^. At the same time, diets that are not nutritionally balanced have contributed to more than a quarter of deaths globally^(2)^ mostly from diet-related chronic diseases requiring costly treatments^(3)^. Many governmental bodies and health authorities now recognise the urgency required to tackle this problem. For example, the UN’s Sustainable Development Goals (SDG) include an aim to achieve food security, improve nutrition and promote sustainable agriculture (i.e. SDG 2)^(4)^. To tackle these concerns, dietary guidelines on environmental sustainability have been published worldwide^(5,6,7,8,9,10)^, and there are an increasing number of recommendations and guidelines that promote a more plant-based diet. In 2019, the EAT-Lancet commission proposed a global healthy reference diet that each country could modify to meet specific nutritional and cultural needs while focusing on environmental sustainability^(11)^. The EAT-Lancet reference diet recommended a predominantly plant-based diet, rich in fruits, vegetables, wholegrains, legumes, nuts and unsaturated oils, with a low-to-moderate amount of seafood and poultry, and a small quantity of red meat, milk, and dairy products. In the UK, Public Health England updated the Eatwell Guide^(12)^ to include the promotion of more plant-based foods (e.g. milk alternatives included in the dairy products section), and the British Dietetic Association (BDA) One Blue Dot policy^(13)^, considers environmental sustainability in the context of healthy, nutritional, diets.

The general recommendation for predominantly plant-based diets and increased use of plant-based alternative products (e.g. milk alternatives) may have the unintended consequence of reducing iodine intake. Plant foods have a low iodine concentration (Table 1), so individuals who follow a plant-based diet may be at risk of iodine deficiency; animal sources (e.g. milk, fish and eggs; Table 1) currently provide the majority of iodine intake in many countries, including the UK^(14)^. In some countries (UK, Ireland and Iceland)^(15,16,17)^, there is no iodised salt policy so moving away from animal foods will have negative impact on iodine intake to a greater degree than in countries where animal foods provide a lower proportion of total iodine intake, and where iodised salt policies exist.

**Table 1.** The iodine content of a range of animal and plant foods by UK recommended portion size

Food	Portion size (g)	Iodine/portion* (µg)
Dairy products and alternatives		
Cows’ milk: whole, semi and skimmed	200	60
Unfortified milk alternatives	200	2
Hard cheese, for example, Cheddar, edam and red Leicester	40	15
Soft cheese, for example, mascarpone and mozzarella	40	8
Yogurt	150	62
Seafood and seaweed		
Cod	120	209
Haddock	120	352
Canned tuna	100	12
Salmon	120	14
Mussels	40 (no shells)	99
Prawns	60	6
Scampi	170	162
Cod fish fingers	60 g (2 fish fingers)	66
Nori	2·5 (2 sheets)	37
Wakame	30	5049
Kombu	5	22 033
Other		
Eggs	100 (2 eggs)	50
Meat and poultry	75	10
Nuts	25	5
Bread: white and brown	2 70 g (2 slices)	10
Fruits and vegetables	80	3

* Values are taken from UK food tables^(19)^.

The FAO of the UN and the WHO launched their guiding principles for sustainable and healthy diets^(18)^. These principles included increasing intake of plant foods such as fruit and vegetables, while including moderate amounts of dairy products, eggs and fish^(18)^. Interestingly, the FAO and WHO report specifically mentions the need to limit salt intake but to ensure that any salt is iodised^(18)^ to ensure adequate iodine intake, and this is especially important in the context of a plant-based diet.

It is unclear whether plant-based dietary patterns, such as the EAT-Lancet reference diet, would provide a sufficient intake of iodine. Unlike other plant-based dietary recommendations, the EAT-Lancet reference diet is quantified, providing guideline daily intakes (average and range in g/d) for each food group. It is therefore possible to calculate the iodine provided, which we have done using UK data, as an example of a country without a salt iodisation programme.

## Scope of this review and methodology

In this narrative review, we have summarised the current published evidence for the risk of iodine deficiency in those following a predominantly plant-based diet (not just a strict vegan diet). The literature review was conducted utilising PubMed, Google Scholar and Cochrane Library databases up until December 2022. Relevant publications were selected using a combination of keywords for plant-based diets and iodine intake (e.g. plant-based, vegan, vegetarian, iodine, intake, status and deficiency). Additional studies were identified by a manual search of bibliographic references in original papers and reviews.

In addition to the narrative review, we estimated the iodine provided by the EAT-Lancet reference diet using iodine concentration data from UK food tables^(19)^. This reference diet consists of food groups from which target intake levels and reference ranges are suggested (e.g. 250 g dairy foods/d with range of 0–500 g). The reference ranges are described by the EAT-Lancet authors as uncertainty ranges and considered to be compatible with optimal health in different populations^(11)^. We calculated the iodine concentration of each food group using the food components listed in the appendices of the EAT-Lancet report^(11)^ and matching this to the corresponding iodine concentration data from UK food tables. A list of the corresponding food components from UK food tables used in this analysis can be found in Supplementary Table 1.

Total daily iodine intake provision from EAT-Lancet reference diet was calculated and compared with iodine intake recommendations for iodine in adults (150 µg/d) and during pregnancy (200–250 µg/d). We used different intake recommendations for comparison, primarily using the values equivalent to the Reference/Recommended Nutrient Intake (RNI) as we calculated provision of iodine from the EAT-Lancet reference diet (in terms of dietary planning^(20)^), not the assessment of population average intakes (for which the Estimated Average Requirement would be more appropriate)^(20,21)^. For adults, we compared iodine provision of the diet against the recommendation of 150 µg/d (the RNI/RDA/Adequate Intake according to WHO^(22)^, USA^(23)^ and European^(24)^ recommendations, respectively) and the UK value of 70 µg/d for the Lower Reference Nutrient Intake (the estimated intake required to prevent goitre)^(25)^. For pregnancy, we present the comparison as a range to account for the variation in iodine intake recommendations around the world, from 200 µg/d in Europe (AI)^(24)^ to 220 µg/d in the USA (RDA)^(23)^ and 250 µg/d according to WHO (RNI)^(22)^.

As a final part of this review, we highlight potential options for those following a plant-based diet to achieve an adequate iodine intake.

## Iodine deficiency as a public health concern

Iodine is essential for synthesising the thyroid hormones, triiodothyronine (T3) and tetraiodothyronine (T4), and an inadequate iodine intake can lead to various adverse effects, collectively termed the iodine deficiency disorders (IDD)^(26)^. Although goitre (enlargement of the thyroid gland) is the most visible effect of iodine deficiency, cognitive impairment has the greatest impact on individuals and populations^(27)^. The negative association between iodine deficiency and cognition is a result of the role that thyroid hormones have in brain and neurological development, meaning that iodine is particularly important during the first 1000 d of life, when the brain is developing^(28)^. A study in the UK has shown that even mild-to-moderate iodine deficiency during pregnancy is associated with lower IQ and reading ability in school-aged children^(29)^.

In recent years, iodine deficiency has re-emerged as a public health concern in women of reproductive age in several European countries^(30,31,32)^, including the UK and Ireland^(33,34,35,36)^. These countries had been considered to be iodine replete for decades, but concern surrounding the re-emergence of iodine deficiency in the UK was ignited in 2011 when the first nationwide survey of iodine status for more than 50 years found mild iodine deficiency in a teenage schoolgirls^(37)^. Since then, several regional studies in the UK and Ireland have reported mild iodine deficiency in adolescent girls and women of childbearing age^(33,34,35,38)^. Results from the UK’s National Diet and Nutrition Survey (NDNS) shows iodine sufficiency in children (4–18 years) and adults (19–64 years), but mild iodine deficiency in women of childbearing age (16–49 years). The data also show a downward trend in iodine status (as measured by urinary iodine concentration) of women of childbearing age from 2013 (when first introduced) to the latest data in 2019^(39)^.

There is concern that pregnant women in the UK are iodine-deficient – although the data are not from the nationally representative NDNS survey (as they are excluded)^(40)^, but from regional studies across the UK^(29,41,42,43,44,45,46,47)^ and the thresholds for defining deficiency during pregnancy are not considered as robust as those in children^(48)^. In other countries of Europe, women are often classified as deficient during pregnancy, even in countries where children are classified as iodine-sufficient^(49)^, and data from dietary surveys show low iodine intake in pregnant women and those of childbearing age^(50)^. Indeed, the focus should be on women of childbearing age, not just in those with confirmed pregnancy. Iodine deficiency in women prior to pregnancy, where thyroidal stores of iodine are not optimised, is increasingly considered to be a risk factor for impaired maternal thyroid hormone profile^(51)^ and may be linked to reduced cognitive outcomes of the child^(52)^. This matters because plant-based diets are more likely to be followed by young women, and data from the UK show that women are more likely to consume alternatives to milk and dairy products than other groups^(53)^.

## Dietary sources of iodine

A plant-based diet is generally considered to be one where plant foods make up the majority of the diet, but there can be inclusion of animal products. A vegetarian, rather than a vegan, diet would be considered as plant-based, as meat and fish are not consumed, but eggs and/or dairy products might be included. However, in recent years, the term has been applied to those who follow a flexitarian style diet – where animal foods may be consumed in limited amounts (including meat and fish), but where most meals are based on plant-based foods and products.

Animal products, including milk, fish and eggs, provide over 60 % of adult iodine intake in some countries (e.g. Iceland, Norway, Spain and the UK) and contribute considerably to total iodine intake (Table 2).

**Table 2.** The contribution of animal products to total iodine intake in adults in the ten European countries that have available dietary data, based on a recent review^(50)^

	Contribution to total iodine intake					Iodised salt policy
	Milk/dairy products (%)	Fish (%)	Egg (%)	Meat (%)	Total animal products (%)	
Belgium	14	7	0	7	28	V
Denmark	30	7	2	1	40	M
Finland	37	10	4	13	64	V
France	21	13	4	5	43	V
Iceland	30	47	2	3	82	N
Ireland	53	6	6	4	58	N
The Netherlands	15	4	2	3	24	V
Norway	36	21	5	3	65	V
Spain	12	32	13	5	62	V
UK	34	10	7	10	61	N

M, mandatory iodised salt policy; V, voluntary iodised salt policy; N, no iodised salt policy.

Table created using data from Bath *et al.* 2022^(50)^.

Table does not show iodised salt policy for all countries in Europe – only those with data on contribution of food groups to total intake in adults.

### Milk and dairy products

Milk and dairy products are the primary sources of iodine in many countries, contributing about 12–53 % of total daily iodine intake (Table 2)^(50)^. Naturally, cows’ milk has a low iodine concentration but has become a rich source of iodine through farming practices, such as providing cattle with iodine-enriched feed and using iodine-based disinfectants known as iodophors during the milking process^(54)^. Countries such as Australia and New Zealand have replaced iodophors with other disinfectants, which may explain the decline in milk iodine concentration in those countries^(55)^. Similarly, the considerable variability in milk iodine content within and between countries is likely a result of differences in farming practices^(54)^. Within some countries (including the UK), there is seasonal variation in milk iodine concentration, where summer milk has a lower concentration than winter milk; this results from greater reliance on iodine-fortified feed during the winter months rather than on pasture grazing^(54)^. In addition, both UK and other European studies have previously shown that organically-produced cows’ milk is lower in iodine compared with conventional cows’ milk^(56)^, mainly due to restrictions on iodine-fortified cattle feed and higher goitrogen components of the cattle feed (e.g. clover-based fodders and feeds^(54)^). Despite this, organic milk is still a good source of iodine, and more recent research suggests no overall difference in iodine concentration between organic and conventional milk in the UK, likely due to changing farming practices in the organic sector^(57)^.

### Milk-alternative drinks

While the EAT-Lancet commission does not explicitly include plant-based milk alternatives in the reference diet, the BDA One Blue Dot policy and the updated Eatwell Guide recommend that milk and dairy products be consumed interchangeably with plant-based alternatives. Notably, these recommendations do not stipulate that consumers should also ensure that these products are fortified with iodine. This is an important point because research in the UK^(58)^, Norway^(59)^ and the USA^(60)^ has shown that unless fortified with iodine, plant-based milk alternatives have a very low iodine concentration – just 2·1 % of the value of UK cows’ milk^(58)^. A 200-g portion of unfortified drinks would provide 0·9–4·3 µg of iodine^(58)^.

In 2015, just 6 % of drinks on the UK market were fortified with iodine. We conducted an updated survey of plant-based alternatives available in the UK in 2020 and found that only 19·8 % of plant-based milk alternatives were fortified with iodine (compared with 63·0 % fortified with Ca); hence, the likelihood of consumers selecting an iodine-fortified product remains low^(61)^. As most of the products available on the UK market are produced abroad, these results are most likely relevant for other European countries.

Dairy products such as cheese and yogurt are also important sources of iodine in the UK (Table 1), and dairy products alone (i.e. excluding the contribution from liquid milk) contribute to 11 % of adults’ and 17 % of children’s iodine intake^(40)^. Data show that consumers are buying less liquid milk and more milk derivatives such as cheese and yogurt^(62,63)^. Non-milk dairy products alternatives, such as plant-based yogurt and cheese, are not usually fortified with iodine^(61)^ and if unfortified plant-based alternatives are used in place of dairy products, iodine intake may be reduced.

It is plausible that adherence to diets that limit milk and milk products and replace them with plant-based alternatives may result in an inadequate iodine intake. A recent study using iodine intake and status (measured as median urinary iodine concentration) from the NDNS (Years 7–9; 2014–2017) found that individuals who exclusively consumed (largely unfortified) plant-based milk alternatives had a lower iodine intake (94 *v*. 129 µg/d) compared with exclusive cows’ milk consumers, as well as a lower iodine status (median urinary iodine concentration: 79 *v*. 132 µg/l), indicating that this group was iodine-deficient according to the WHO threshold for population sufficiency (>100 µg/l)^(53)^. The study found that plant-based milk alternatives were more likely to be consumed by young women (7·6 % of women 16–49 years *v*. 4·6 % of the total NDNS sample were milk-alternative consumers)^(53)^. This is a concern as young women who consume diets low in iodine are especially vulnerable to maternal and fetal effects of iodine deficiency if they become pregnant.

### Eggs

Chicken eggs are a good source of iodine, with a concentration of 50 µg/100 g, or approximately 25 µg of iodine provided by one egg (Table 1). Eggs are estimated to contribute between 2 % and 13 % of total iodine in Europe countries^(50)^. Though data on the variability in egg iodine content are limited, it is believed to be affected by factors such as feed content, the physical status of hens such as age and laying rate, and environment^(64,65)^.

### Fish

Fish and seafood products are naturally rich sources of iodine, although absolute levels are species-specific, creating a broad range of food options and iodine provision per portion (Table 1). Fish consumption in the UK is low, and therefore it makes a smaller contribution to population iodine status than milk and dairy products, contributing 11 % of the adult intake^(66)^. However, on a per-portion basis, fish has the potential to contribute significantly towards iodine intake recommendations.

The current UK recommendation for fish and seafood includes eating two 140 g portions per week (280 g fish per week or 40 g/d), one of which should be from oily fish^(12)^. According to NDNS data^(66)^, UK adults are currently not meeting this recommendation, with a median daily intake of total fish at 13 g/d (median 0 g/d of oily fish^(66)^, reflecting the high proportion of non-consumers). By contrast, Nordic dietary patterns emphasise fish intake, and seafood is consumed more regularly in those countries. The adult mean daily fish intake is higher in Norway (67 g/d^(67)^) and contributes more to iodine intake. Indeed a recent study found that only women aged 18–35 years in Norway had a fish intake below the recommended two portions per week^(68)^. Consequently, implementing plant-based dietary recommendations in countries with a high fish intake, such as Norway, may decrease iodine intake.

There is a potential conflict between dietary recommendations for fish intake and sustainability considerations, as an increase in global fish intake may put additional strain on global fish stocks. The EAT-Lancet commission combats this by recommending consumers choose farmed seafood rather than wild-caught to conserve wild fish populations. However, iodine-rich fish such as cod and haddock are not widely farmed^(69)^, and therefore a diet prioritising farmed seafood will provide less iodine than one without this restriction, even if the recommended portions are consumed. Additionally, aquaculture production has a range of environmental concerns of its own. Increased aquaculture production will result in significant resource constraints due to limited freshwater resources^(70)^. There is also widespread irreversible habitat destruction due to aquaculture which is likely to affect biodiversity negatively^(71)^.

To guide consumers in making an environmentally responsible choice, the Marine Conservation Society has developed their Good Fish Guide^(72)^, which rates seafood based on its sustainability and where and how it is caught. Approximately 80 % of all seafood sold in the UK is from five species (cod, haddock, salmon, tuna and prawns)^(72)^. Of these five fish species, Atlantic cod, Atlantic salmon, tuna and prawns all received a mixed rating, and consumers are recommended to look for more sustainable alternatives. However, haddock and Pacific cod are currently rated as good options, both of which are rich sources of iodine and so should be encouraged in those striving to meet the dual goals of iodine sufficiency and diet sustainability.

### Seaweed

Seaweed is often cited as a rich plant-based source of iodine; however, the iodine content is highly variable and unreliable as a source of iodine and could even result in excessive iodine intake^(73,74,75,76,77,78)^. For example, kelp has a high iodine concentration (Table 1); therefore, even a 5-g portion could provide 22 033 µg of iodine, over 14 000 % of the adult recommended intake^(22)^, well in excess of the safe upper limit of 600 µg/d^(79)^. Indeed, in a sub-national study in Denmark, researchers found that regular seaweed consumers had a high or excessive iodine intake (>900 µg/d)^(80)^. Kelp supplements should not be used as a source of iodine, and regular brown seaweed intake is not recommended^(81)^. However, not all seaweed species have very high iodine content, and species with an appropriate iodine concentration might be able to reduce the risk of iodine insufficiency in those following a plant-based diet. Seaweed is gaining popularity as a functional ingredient in plant-based products to enhance taste or food matrix or to enrich the product with bioactive compounds^(82,83,84,85)^. However, additional research is required to understand the contribution of seaweed to iodine intake and whether this is a safe source of iodine, given the potential for toxicity. In addition, the bioavailability of iodine from seaweed is not well understood^(86)^.

### Iodised salt as a source of iodine

To prevent and control iodine deficiency disorders, the WHO recommends the iodisation of all food-grade salt^(22)^. Consequently, many countries such as Austria, Denmark, Italy and Turkey have enacted mandatory fortification of salt (Table 2)^(50)^. However, only 40 % of European countries have a mandatory salt iodisation policy^(50)^, with countries including the UK and Ireland having no such requirement. Therefore, there is a lack of iodine fortification in the UK and Ireland, and the availability of iodised salt is low^(15,16)^. As a result, iodine status in these countries depends on individual food choices, with milk and dairy products often the primary dietary source. For example, milk and dairy products in the UK provide 51 % and 33 % of children’s and adult’s iodine intake, respectively^(14)^. Other countries are similarly heavily reliant on appropriate food choices in the absence of iodine fortification policies, for example, in Norway, where, although officially noted as having a voluntary iodised salt policy, the permitted iodine content of salt is low, and there is limited use an availability of iodised salt in the country^(32)^.

Other countries, such as the Netherlands, Denmark and Belgium, have fortified bread with iodine through the use of iodised salt in bread making^(87,88,89)^. This provides a non-animal source of iodine in those countries. Indeed, 49 % and 53 % of adult iodine intake in Belgium and the Netherlands are from bread and cereals due to the iodine fortification of bread^(50)^. By contrast, the contribution of bread and cereals is low in countries where bread is not fortified with iodine; for example, it provides just 6 % and 12 % of total iodine intake in Norway and the UK, respectively^(50)^.

## Vegan diets and iodine intake

It is estimated that between 2 and 3 % of the UK population are following a vegan diet^(90,91)^, with 620 000 participating in ‘Veganuary’ (the pledge to follow a vegan diet in January) in 2022, a 268 % increase from 2018^(92,93)^. Vegan diets, where there is complete removal of animal products from the diet, provide an example of the effect of strict plant-based diets on iodine intake. Although the scientific evidence in this regard is relatively scarce, and the sample sizes of most studies are insufficient to draw a definitive conclusion, available evidence shows that those following a vegan diet are at high risk of iodine deficiency^(94,95,96,97)^.

Three reviews have investigated the iodine status of those consuming vegan and vegetarian diets^(94,98,99)^. The most recent review published in 2020^(94)^ identified fifteen relevant studies that examined iodine intake or status by different dietary groups such as vegetarian, vegan, omnivore and lacto-vegetarian; the data show that adults following a vegan diet (not taking an iodine-containing supplement) have an increased risk of low iodine status, iodine deficiency and inadequate iodine intake than those following an omnivorous diet^(94)^. It should be noted that several of the studies included in the analysis did not measure the iodine intake from salt, and any iodine from dietary supplements was often excluded from the dietary intake. Notably, the highest iodine intake was recorded in vegans who habitually consume seaweed, with intakes well above the RNI. In these circumstances, sudden and sustained excess of iodine can inhibit hormonal biosynthesis (Wolff–Chaikoff effect)^(100,101)^. This inhibition is typically transient and of short duration if iodine intake returns to the recommended range. However, prolonged excess intake of seaweed, such as kelp, a brown seaweed often called kombu, has been linked to cases of thyroid dysfunction, including both hyper- and hypothyroidism^(102)^.

There are case reports of goitre (i.e. a symptom of severe iodine deficiency) in those following a vegan diet, resulting in a severely low iodine intake, including cases in a UK neonate (as a result of maternal vegan diet in pregnancy)^(103)^, toddler in the USA^(104)^, children in the UK^(105)^ and UK women of childbearing age^(106)^. In addition, there are individual case reports that have highlighted the risk of severe iodine deficiency in those following a restrictive diet as a result of medical conditions, such as cows’ milk allergy^(107,108)^ or other reasons^(109)^. Finally, a randomised controlled trial in Sweden found that adults following a palaeolithic diet for 2 years (that restricted salt and dairy product intake) had a higher risk of iodine deficiency (as measured by urinary iodine concentration) than those on a diet following Nordic Nutrition Recommendations^(110)^. These studies all underline the susceptibility to iodine deficiency in those following a diet restricting the consumption of iodine-rich foods.

## Iodine provided by predominantly plant-based diets

While the effect of a strict vegan has been explored in relation to iodine intake, and risk of iodine deficiency, less is known about a more moderate following of a plant-based diet. We therefore calculated the iodine provision of the EAT-Lancet reference diet, which recommends a moderate intake of dairy products, and limited meat, fish, and egg intake, with plenty of fruit and vegetables. The specific quantities of iodine-rich foods recommended by the EAT-Lancet reference diet (Table 3) are daily intake of 250 g dairy products (i.e. both milk and dairy products, range 0–500 g), 28 g fish (196 g/week) and 13 g egg (approximately 1·5 eggs/week)^(11)^.

**Table 3.** The EAT-Lancet reference diet, with recommended daily food intake (including possible ranges) for an adult consuming 2500 kcal/d with estimated iodine content of each food group per 100 g/d, and contribution to WHO recommendation for iodine intake^(22)^

	Food intake (possible) g/d	Range	Iodine content µg/100 g	Iodine provision µg/d	Range	Contribution to adult intake recommendation of 150 µg/d (%)^(22,23,24)^	Range
Wholegrains							
Rice, wheat, maize and other	232	-	0	0	-	0	-
Tubers or starchy vegetables							
Potatoes and cassava	50	0–100	0	0	0–0	0	0–0
Vegetables							
All vegetables	300	200–600	2	6	4–12	4	3–9
Dark green vegetables	100		2	2		1	
Red and orange vegetables	100		1	1		1	
Other vegetables	100		3	3		2	
Fruits							
All fruit	200	100–300	2	4	2–7	3	2–5
Dairy foods							
Whole milk or derivative equivalents (e.g. cheese)	250	0–500	31	76	0–153*,†	51	0–102
Protein sources							
Beef and lamb	7	0–14	3	0	0–0·4	0	0–0
Pork	7	0–14	0	0·0	0–0	0	0–0
Chicken and other poultry	29	0–58	5	2	0–3	1	0–2
Eggs	13	0–25	50	6	0–13	4	0–8
Fish	28	0–100	88	25	0–88	16	0–58
Legumes							
Dry beans, lentils and peas	50	0–100	3	2	0–3	1	0–2
Soya foods	25	0–50	0	0	0–0	0	0–0
Peanuts	25	0–75	19	5	0–14	3	0–10
Tree nuts	25		7	2		1	
Added fats							
Palm oil	6·8	0–6·8	0	0	0–0	0	0–0
Unsaturated oils	40	20–80	0	0	0–0	0	0–0
Dairy fats (included in milk)	0		38	0		0	
Lard or tallow	5	0–5	0	0	0–0	0	0–0
Added sugar							
All sweeteners	31	0–31	0	0	0–0	0	0–0
Total iodine intake (µg/d)	–		–	128	8–295*,†	85	6–196

* If using unfortified milk alternative (at 1 µg/100 g), iodine provision from dairy category would be 2·5 µg/d (range 0–5µg/d) and the total daily iodine intake would be 54 µg/d (range 8·3–147 µg/d) or 36 % (5·5–98 %) of adult intake recommendations.

† If using fortified milk alternative (at 22·5 µg/100 g), iodine provision from dairy category would be 56·25 µg/d (range 0·00–5) and the total daily iodine intake would be 108 µg/d (range 8·25–255 µg/d) or 72 % (5·5–170 %) of adult intake recommendations.

Following the diet would result in an estimated daily iodine intake of 128 µg/d (Table 3), which is 85 % of the recommended intake for adults (at 150 µg/d^(22,23,24)^); as the provision of iodine is below the RNI, the distribution of intake in a population would mean that is likely that a high proportion of individuals would have low intake (i.e. intake below the Estimated Average Requirement or Lower Reference Nutrient Intake). When considering the range of intake suggested for each food category, the iodine provision could be as low as 8 µg/d (6 % of adult RNI) or as high as 295 µg/d (196 % of daily adult RNI). However, the higher end of the range includes 100 g of fish and 500 g of dairy products per d which does not line up with the overall message of the EAT-Lancet commission to have a low-to-moderate intake of animal-based products. The calculated provision of iodine in the EAT-Lancet reference diet, at 128 µg/d, is below the RNI for pregnancy – the diet would provide just 51–64 % of the pregnancy recommended intake of iodine (200–250 µg/d)^(22,23,24)^ and therefore would place individuals at risk of iodine deficiency during pregnancy. The calculation of iodine provision is based on food sources only, and we recognise that in countries with iodised salt programmes, additional iodine may be provided through iodised table salt use, although this would be unlikely in the UK^(15)^ or other countries such as Norway.

It is clear from Table 3 that the majority of iodine in the EAT-Lancet reference diet is from the dairy foods category (250 g of cows’ milk would provide 76 μg/d, or 51 % of the WHO recommended iodine intake for adults). When considering the range of potential dairy product intake (0–500 g/d), the higher end of dairy product intake would provide a sufficient iodine for an adult (500 g of UK milk would provide an estimated 150 μg/d). However, the overall message diet, and plant-based diets, in general, is to reduce the consumption of foods of animal origin. Indeed, the commission suggests that increasing milk consumption from 250 g to 500 g would increase greenhouse gas emissions and total environmental effects regardless of changes to production practices. Consequently, the commission recommends that the optimal intake of dairy products should be at the lower end of the reference range which will lower the overall iodine content of the reference diet. To meet this recommendation, some may turn to plant-based milk alternatives and if cows’ milk is replaced with a fortified plant-based alternative (fortified at 22·5 µg/100 ml, the most commonly used level of iodine fortification^(61)^), the reference diet would provide a total of 108 µg iodine/d, 72 % of the adult RNI and 43–54 % of iodine intake recommendations in pregnancy^(22,23,24)^. However, it is far more likely that consumers would replace cows’ milk with an unfortified milk alternative, as the majority of the products on the market do not add iodine to replace that found in cows’ milk^(61)^. We estimate that 250 g of a plant-based milk alternative not fortified with iodine (estimated iodine concentration = 1 µg/100 g) would provide just 2·5 µg iodine/d (Table 3). Therefore, consumers replaced cows’ milk with a unfortified plant-based milk alternative the diet would provide a total of just 54 µg iodine/d, 35 % of the adult RNI and 22–27 % of the RNI in pregnancy. The provision of just 54 µg iodine/d is below the Lower Reference Nutrient Intake for adults (of 70 µg/d) which was set as the intake required to prevent goitre^(25)^. Therefore, without adequate advice on supplementation, individuals following the EAT-Lancet reference diet using unfortified milk alternatives would be at high risk of deficiency, and if this was during pregnancy this would be of public health concern in terms of risk to fetal neurodevelopment^(111)^.

The egg category provides 6 μg of iodine per d (4 % of adult RNI; Table 3). The average UK consumer consumes 2·9 eggs per week^(112)^, not including the eggs added to prepared food products; therefore, adhering to the diet would necessitate a reduction in egg consumption and, therefore, in iodine provision from this food source in the UK.

The fish category provides an estimated 25 µg iodine/d (Table 3), based on the iodine content of the fish examples provided in the EAT-Lancet report (one portion (98 g) of sockeye salmon and one portion (98 g) of Atlantic cod per week)). However, if the consumer chose alternative fish sources, the iodine provision from fish would be much lower – 196 g of salmon or canned tuna per week would provide just 4 and 2 μg/d, respectively. This highlights that the iodine provision is highly dependent on the species of fish consumed, as well as the overall quantity. The commission recommend that oily fish is chosen in preference as it provides greater quantities of *n*-3 fatty acids; however, white fish has a higher iodine concentration than oily fish (Table 1). While some plant-based diets may exclude fish consumption altogether, the amount of fish recommended by the EAT-Lancet commission is higher than usual intake in the UK (13 *v*. 28 g/d). However, encouraging people to meet the recommendation of 196 g/week may be challenging in the UK with consumer trends indicating that fish consumption is already declining^(14)^.

There are several caveats regarding the calculation of iodine in the EAT-Lancet reference diet. Firstly, there is the variation in milk iodine concentration, both by farming type (organic or conventional) and by season. This makes it difficult to determine the exact iodine provision by the EAT-Lancet reference diet. In the summer months, a 250-g portion of UK milk would provide 50 μg of iodine compared with 103 μg in winter, thereby changing the total iodine provided from 102 to 155 μg/d. Secondly, we calculated the contribution of milk and dairy products to iodine intake using values for liquid milk, which may underestimate the amount of iodine provided by the reference diet, as other dairy products such as certain types of cheese may have a higher iodine concentration (on a per gram basis) than milk. For example, while Cheddar cheese (30 μg/100 g) provides a similar amount of iodine to cows’ milk, halloumi (60 μg/100 g) and red Leicester cheese (46 μg/100 g) would contribute more to overall intake. However, the majority of dairy product intake will likely come from milk^(62)^, and therefore this is unlikely to affect the overall iodine content of the EAT-Lancet reference diet. The iodine content of the EAT-Lancet reference diet was calculated using UK data with the assumption of no iodised table salt, as the UK does not have a universal salt iodisation programme and the availability of iodised salt is low^(113)^. Finally, the iodine provision may be lower than we calculate as the quantities of food recommended by the commission are based on calorie requirements (2500 kcal/d) of a 70-kg male or a 60-kg female (aged 30 years) whose physical activity level is moderate to high. If quantifies are adjusted to meet 2000 kcal/d instead, it is likely that the recommendations for animal-based products would be lower, and consequently, so would the iodine provided.

## The narratives and perceptions of plant-based diets concerning iodine intake

Iodine is often overlooked when it comes to dietary recommendations. The EAT-Lancet commission does not mention iodine in its report and does not consider it an essential micronutrient when creating the reference diet. The dairy product recommendation is primarily based on Ca intake, and the fish recommendation is based on *n*-3 fatty acids. However, it is not just the EAT-Lancet reference diet that overlooks iodine. Many plant-based dietary recommendations focus on Ca when discussing dairy products and plant-based alternatives. UK-based recommendations such as the Eatwell Guide and the BDA One Blue Dot policy both recommend that Ca-fortified plant-based milk alternatives can be consumed interchangeably with cows’ milk without mentioning iodine. As plant-based diets continue to gain popularity, the lack of attention given to iodine will become an even greater issue.

There is inaccurate information found online (e.g. popular websites and social media) about plant-based sources of iodine. While The Vegan Society (UK) has accurate information on iodine on their website^(114)^, other information online (including across social media) often incorrectly cites plant-based food sources as being rich in iodine – including particular fruits (e.g. prunes, strawberries or cranberries), potato skins, and navy or kidney beans^(115,116)^. The iodine content of plant crops is affected by the content of iodine in the soil, but, in general, plant-based foods such as vegetables and fruits are relatively poor sources of iodine^(117)^. Based on UK food table data, strawberries contain just 1 µg/100 g, dried prunes (100 g or 10 prunes) would contribute only 2 µg^(19)^, potatoes contain approximately 1 µg/100 g and a 400-g can of kidney beans would provide approximately 12 µg of iodine^(19)^. It is important to counter these myths around plant sources of iodine, where possible, and provide suitable and safe sources of iodine to those following a plant-based diet.

The fact that the EAT-Lancet reference diet provides 51–64 % of the iodine intake recommended in pregnancy, or just 22–27 % if an unfortified milk alternative is chosen, is of concern and should be highlighted to consumers. It is particularly notable because the EAT-Lancet reference diet includes a recommendation for pregnant women to supplement their diet with vitamin B_12_, as it is acknowledged that the diet does not provide enough B_12_ for pregnancy. The EAT-Lancet commission does not mention iodine supplementation in pregnancy, but our analysis highlights that the diet, without adequate iodine supplementation, would not provide sufficient iodine, especially if plant-based alternatives to cows’ milk were consumed. We acknowledge that there is a degree of debate around the recommended iodine intake in pregnancy^(50)^, and whether needs can be met if thyroid stores are optimised prior to pregnancy^(118)^; however, the EAT-Lancet reference diet may not provide enough iodine for women of childbearing age so that thyroidal iodine stores can be maintained, particularly if unfortified milk alternatives are used in place of cows’ milk.

## Options for those following a plant-based diet to optimise iodine intake

It is important to note that plant-based diets and iodine sufficiency do not have to be mutually exclusive. For those following a plant-based diet, there are ways to optimise iodine intake. For example, if avoiding milk, individuals should opt for iodine-fortified plant-based alternatives or increase their iodine intake from other food sources. A suitable iodine-containing supplement would be a more consistent and reliable way of ensuring an adequate iodine intake if this cannot be achieved through the diet. If a supplement is considered, this should not be a seaweed supplement; instead, iodine should be in the form of potassium iodide or potassium iodate with a dose that does not exceed 150 µg/d.

### Conclusion

Although dietary recommendations, such as the EAT-Lancet reference diet, provide a valuable roadmap for healthy, sustainable diets, this review shows that they may not provide enough iodine to meet adult or pregnancy intake recommendations. With an overarching message to reduce animal product intake, consumers and health professionals must be aware that plant-based diets may exacerbate dietary and nutritional inadequacies such that of iodine, unless there is clear guidance for the consumer on use of fortified foods or appropriate supplementation. As evidenced in this review, those transitioning to a plant-based diet in the UK, a country without widespread use of iodised salt, may be at risk of iodine deficiency.

## Supplementary material

For supplementary material accompanying this paper visit https://doi.org/10.1017/S0007114523001873. click here to view supplementary material
