# Supplementary material for: Iodine and plant-based diets: a narrative review and calculation of iodine content
Source: Br J Nutr. 2023 Aug 25;131(2):265–75. doi: 10.1017/S0007114523001873 (PMC10751939; doi:10.1017/S0007114523001873)
Supplement: Supplementary file 1 [file S0007114523001873sup001.docx]

**Supplementary Table for “Iodine and plant-based diets – a narrative review and calculation of iodine content”**

**Authors:** K. Nicol^1^, A.P. Nugent^2^, J.V. Woodside^3^, K. H. Hart^1^, S.C. Bath^1^

**Supplementary Table 1**. Food codes from UK food tables used in the calculation of iodine content in the EAT-Lancet.

| Code | Food Name | Iodine (µg/100g) | EAT-Lancet group |
| --- | --- | --- | --- |
| 18-469 | Beef, mince, raw | 9 | Protein sources |
| 13-062 | Beans, blackeye, whole, dried, raw | 0 | Legumes |
| 13-657 | Lentils, red, split, dried, raw | 6 | Legumes |
| 18-301 | Chicken, corn-fed, raw, meat only | 5 | Protein sources |
| 12-598 | Milk, whole, pasteurised, summer and autumn | 20 | Dairy foods |
| 12-597 | Milk, whole, pasteurised, winter and spring | 41 | Dairy foods |
| 17-685 | Butter, salted | 38 | Added fats |
| 12-937 | Eggs, chicken, whole, raw | 50 | Protein sources |
| 16-373 | Cod, flesh only, baked | 161 | Protein sources |
| 16-359 | Salmon, farmed, flesh only, baked | 14 | Protein sources |
| 14-319 | Apples, eating, raw, flesh and skin | 4 | Fruits |
| 14-347 | Bananas, raw, flesh only, weighed with skin | 2 | Fruits |
| 14-327 | Oranges, flesh only | 1 | Fruits |
| 17-010 | Lard | 0 | Added fats |
| 14-878 | Peanuts, dry roasted | 19 | Legumes |
| 18-510 | Pork, fillet medallions, raw, lean and fat | 0 | Protein sources |
| 13-570 | Tofu, soya bean, steamed | 0 | Legumes |
| 17-063 | Sugar, white | 0 | Added sugar |
| 13-618 | Potatoes, new and salad, flesh only, raw | 0 | Tubers |
| 14-897 | Almonds, toasted | 2 | Legumes |
| 14-811 | Cashew nuts, kernel only, plain | 11 | Legumes |
| 17-038 | Oil, olive | 0 | Added fats |
| 17-040 | Oil, peanut (groundnut) | 0 | Added fats |
| 17-045 | Oil, sunflower | 0 | Added fats |
| 13-496 | Carrots, old, raw | 0 | Vegetables |
| 13-627 | Courgette, raw | 5 | Vegetables |
| 13-499 | Onions, raw | 2 | Vegetables |
| 13-318 | Peppers, capsicum, green, raw | 1 | Vegetables |
| 13-572 | Spinach, mature, raw | 2 | Vegetables |
| 13-644 | Squash, butternut, baked | 3 | Vegetables |
| 13-519 | Tomatoes, cherry, raw | 2 | Vegetables |
| 11-868 | Rice, brown, basmati, raw | 0 | Whole grains |
| 11-904 | Wheat, bulgur, raw | 0 | Whole grains |
